# Frequent Seronegative Primary Hypothyroidism in Myxedema Coma in Japan: Three Case Reports With a Systematic Review

**DOI:** 10.1155/2024/2524019

**Published:** 2024-10-14

**Authors:** Yosuke Okuno, Kosuke Mukai, Yuri Tamura, Tomoaki Hayakawa, Atsunori Fukuhara, Iichiro Shimomura

**Affiliations:** ^1^Department of Metabolic Medicine, Osaka University Graduate School of Medicine, Osaka, Japan; ^2^Department of Adipose Management, Osaka University Graduate School of Medicine, Osaka, Japan

## Abstract

Myxedema coma is a rare life-threatening form of hypothyroidism that manifests as neuropsychiatric, metabolic, respiratory, and cardiovascular dysfunction. From 2010 to 2022, our hospital managed three cases of myxedema coma. While the overall characteristics of these cases were similar to those in previous reports, we noticed that all cases were negative for thyroid autoantibodies and an autopsy in one of the cases exhibited end-stage thyroiditis. During a systematic review of cases from 1999 to 2022, we also noticed that a significant proportion of myxedema coma was caused by seronegative primary hypothyroidism especially in Japan.

## 1. Introduction

Myxedema coma is a life-threatening form of hypothyroidism that manifests as neuropsychiatric, metabolic, respiratory, and cardiovascular dysfunction. The in-hospital mortality rate is 29.5% in Japan [1] and 25%–60% in other countries [2]. The occurrence of myxedema coma is rare, with an estimated incidence of 1.08 per million people per year in Japan [1] and 0.22 per million per year in Europe [3]. Due to its rarity, we can only obtain the detailed characteristics of myxedema coma from isolated case reports or case series.

From 2010 to 2022, our hospital managed three cases of myxedema coma. All cases were negative for thyroid autoantibodies, and an autopsy in one of the cases represented terminal stage of thyroiditis. We describe these cases in detail, present a systematic review of the literature, and discuss the possible relationship between myxedema coma and thyroid autoantibodies case presentation.

Three patients were diagnosed as myxedema coma at the tertiary emergency medical center of Osaka University Hospital from 2010 to 2022.

## 2. Case 1

In March 2018, an 80-year-old Japanese man was brought to a community hospital due to impaired consciousness. He was treated for type 2 diabetes and Lewy body dementia. Two months prior, he had exhibited primary hypothyroidism (TSH [thyroid-stimulating hormone] 16.53 μIU/ml and fT4 [free T4] 0.86 ng/dl) in another clinic but had not been treated. On admission, his body temperature was 26°C, systolic blood pressure was 70 mmHg, and heart rate was 20 beats per minute (bpm). He presented with hypercapnia (arterial pCO_2_ 53.7 mmHg) and elevated creatine kinase levels (917 U/l [50–230 U/l]). Myxedema coma was considered, and the patient was transferred to a tertiary emergency medical center at Osaka University Hospital. At the time, his Glasgow coma scale (GCS) score was E4V3M5. TSH was 34.33 μIU/ml (0.45–3.72 μIU/ml), fT4 was 0.6 ng/dl (0.8–1.7 ng/dl), and fT3 was 1.0 pg/ml (2.1–3.1 pg/ml). Antithyroid peroxidase antibody (TPOAb) and antithyroglobulin antibody (TgAb) tests were negative. His thyroid gland exhibited atrophy in the plain CT (computed tomography). He did not take any medications known to cause hypothyroidism, and there was no evidence of previous exposure to high-dose iodine. The patient presented sepsis due to methicillin-sensitive *Staphylococcus aureus* from the cellulitis of the left hand and the right knee. The patient also had a chronic subdural hematoma with a midline shift. He was diagnosed with myxedema coma and treated with levothyroxine, together with burr hole evacuation, catecholamines, ventilation, and antibiotics. He was subsequently moved to a rehabilitation hospital on day 22 of admission.

## 3. Case 2

**Figure 1:**
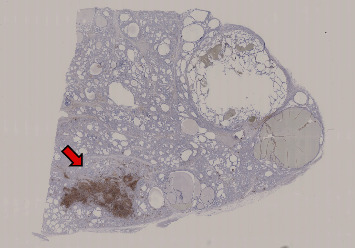
The section from thyroid gland of Case 2 was immunostained using antibody against leukocyte common antigen (LCA). Arrow; local infiltration of lymphocytes.

In June 2018, an 88-year-old Japanese man was brought to the tertiary emergency medical center of Osaka University Hospital because of impaired consciousness at home. The patient had been treated for hypertension. On admission, the GCS score was E1V1M4, and his temperature was 34.4°C. Blood pressure and heart rate were 105/65 mmHg and 65 bpm, respectively. He also exhibited severe hypercapnia (arterial pCO_2_, 150 mmHg). After intubation, his GCS score rapidly improved to E4VTM5. He exhibited primary hypothyroidism (TSH 40.42 μIU/ml [0.45–3.72 μIU/ml], fT4 0.1 ng/dl [0.8–1.7 ng/dl], and fT3 0.9 pg/ml [2.1–3.1 pg/ml]). Tests for TPOAb, TgAb, and anti-TSH receptor antibodies (TRAb) were negative. His thyroid gland exhibited enlargement in the plain CT. Thus, he was diagnosed with myxedema coma and treated with levothyroxine. However, he developed sepsis and renal failure, and died from rupture of an infectious aortic aneurysm on day 88 of admission. An autopsy of the thyroid gland revealed prominent dilated follicles, atrophic follicles, and fibrosis. Lymphocyte infiltration was observed only in a small portion of the thyroid gland (Figure 1).

## 4. Case 3

In February 2022, an 80-year-old Japanese man was brought to the tertiary emergency medical center of Osaka University Hospital because of impaired consciousness at home. He had undergone pancreatoduodenectomy for gallbladder cancer and unilateral total nephrectomy for renal pelvic cancer. He was treated for heart failure with preserved ejection fraction (HFpEF) and chronic kidney disease. He also had untreated chronic obstructive pulmonary disease (COPD). On admission, his GCS score was E3V2M5, and his rectal temperature was 27.1°C. Systolic blood pressure and heart rate was 85 mmHg and 35 bpm, respectively. The patient was intubated for hypercapnia (arterial pCO_2_ 61 mmHg). Although he exhibited only mild to moderate primary hypothyroidism without treatment until six months prior (TSH 6.67 μIU/ml [0.45–3.72 μIU/ml] and fT4 1.4 ng/dl [0.8–1.7 ng/dl] 30 months prior, 6.33 μIU/ml [0.45–3.72 μIU/ml] and fT4 1.7 ng/dl [0.8–1.7 ng/dl] 24 months prior, 12.12 μIU/ml [0.61–4.23 μIU/ml], and fT4 1.0 ng/dl [0.8–1.7 ng/dl] 6 months prior), he presented with severe primary hypothyroidism (TSH 139.15 μIU/ml [0.61–4.23 μIU/ml], fT4 0.2 ng/dl [0.8–1.7 ng/dl], and fT3 1.4 pg/ml [2.1–3.1 pg/ml]) on admission. His TPOAb, TgAb, and TRAb levels were negative. His serum immunoglobulin G4 (IgG4) was normal (102 mg/dL [11–121 mg/dl]). His thyroid gland did not exhibit abnormal finding in the plain CT. He did not take any medications which are known to cause hypothyroidism. The chest CT exhibited COPD, cardiomegaly, pleural effusion, and pulmonary edema, but no pneumonia finding (Supporting Information 1: Figure S1). Antibiotics were initiated for elevated C-reactive protein (CRP) (5.41 mg/dl) and neutrophil-to-lymphocyte ratio (neutrophil 90.3% and lymphocyte 7.3%) together with the history of COPD and decreased SpO_2_. Later, white blood cell and CRP transiently rose to 15,420/μl and 13.26 mg/dL, respectively and *Klebsiella pneumoniae* was identified in the sputum, which were improved by the treatment of antibiotics. He was diagnosed with myxedema coma likely precipitated by upper respiratory tract bacterial infection, treated with levothyroxine, and transferred to a rehabilitation hospital on day 70 of admission.

The characteristics of these cases and the diagnostic scores for myxedema coma [4] are summarized in Table 1.

## 5. Discussion

The characteristics of myxedema coma in our cases were mostly consistent with the literature. All cases were elderly, two patient had an underlying precipitating factor (Cases 1 and 3), and two cases developed in winter or early spring (Cases 1 and 3). However, all three patients were male despite the predominance of myxedema coma in females [2].

Most cases of primary hypothyroidism are caused by Hashimoto's thyroiditis, which is positive for TPOAb and TgAb in 90% and 50% of cases, respectively [5–7]. However, all our patients were negative for TPOAb and TgAb. To our knowledge, no study has investigated the possible relationship between myxedema coma and thyroid autoantibodies. Therefore, we systematically reviewed published case reports and case series of myxedema coma caused by primary hypothyroidism (Supporting Information 2: Table S1). We searched articles in PubMed written in English or Japanese with titles containing “myxedema (myxoedema) coma,” “myxedema (myxoedema) crisis,” or “hypothyroid crisis” from 1999 to 29th June, 2022. Secondary hypothyroidism, discontinuation of levothyroxine, primary hypothyroidism in infants, and primary hypothyroidism with known causes (e.g., immunotherapy-related thyroiditis, drugs [amiodarone/lithium], iron, food [raw bok choy], thyroid tumor, congenital diseases including Down syndrome, and history of thyroidectomy, neck radiation, or isotope therapy) were excluded. Among 108 patients in 52 reports, three cases were negative for TPOAb and TgAb [8–10]. Two cases were negative for TPOAb, but TgAb was not described [11, 12]. Sixteen patients tested positive for TPOAb or TgAb. Thyroid autoantibodies in the remaining 87 cases were not described. Interestingly, all cases negative for TPOAb and TgAb were reported from Japan [8–10], which accounted for 42.9% of the total seven cases in Japan available for thyroid autoantibodies. Including our three cases, of the 10 Japanese cases in the literature for which antibody status was described, 60% were seronegative. On the other hand, only two cases (14%) from USA (United States of America) [11, 12] were negative for TPOAb among cases for which antibody status was described in other countries. The frequency of seronegativity in myxedema coma with primary hypothyroidism was significantly higher in Japan compared with other countries (*p*=0.0192; Table 2). Therefore, seronegative hypothyroidism might be predominant in myxedema coma in Japan, although it may not be generalizable to other countries.

A possible explanation of frequent seronegativity in myxedema coma is that a long-time course of hypothyroidism is usually required for the development of a myxedema coma [13]. In Hashimoto's thyroiditis, TPOAb levels often decrease and sometimes normalize over time. For example, mean TPOAb levels decreased by 70%, and 16% of patients became seronegative during the 5-year follow-up in a study by Schmidt et al. [14]. TPOAb levels also decrease to nearly normal levels after total thyroidectomy in Hashimoto's thyroiditis [15]. Even though the thyroid autoantibodies in our cases might have been positive at the time of the development of Hashimoto's thyroiditis, the antibodies might have normalized after their thyroids had become almost functionally athyreotic, seen in the autopsy in Case 2 (Figure 1).

Another possibility was that our cases were based on the etiologies other than Hashimoto's thyroiditis, including atrophic thyroiditis, IgG4-related thyroiditis, infectious thyroiditis, and drug-related hypothyroidism. Case 1 could not exclude atrophic thyroiditis because his thyroid gland exhibited atrophy in CT, but it was unlikely in Cases 2 and 3 because autopsy from Case 2 exhibited destructive thyroiditis and TRAb was negative in Case 3. Cases 1 and 2 could not exclude IgG4-related thyroiditis, but it was unlikely in Case 3 as he exhibited normal level of serum IgG4. Infectious thyroiditis was unlikely in any case because CT did not exhibit findings characteristic of infectious thyroiditis, such as abscess formation or edema of hypopharynx [16]. Although we did not identify any medications known to cause hypothyroidism, excessive intake of iodine could not be ruled out because we did not evaluate urinary iodine excretion. Although precise etiology of myxedema coma caused by seronegative primary hypothyroidism in our cases needs additional information, our finding would draw attention for measuring thyroid autoantibodies in myxedema coma.

In the Cases 1 and 3, mild to moderate hypothyroidism were already pointed out but left untreated. The current report might suggest that some proportion of mild to moderate hypothyroidism need to be treated properly, if thyroid autoantibodies were negative in the population at high risk for myxedema coma, such as the elderly and the lean.

There is a limitation in our study. As noted above, there are many cases in which thyroid antibody is unknown. The description of thyroid antibody was significantly more frequent in the reports from Japan than those from other countries (*p*=0.0006, Supporting Information 3: Table S2). This difference is subject to selection bias and the results from Table 2 may not reflect the population as a whole.

## 6. Conclusion

All three cases of myxedema coma we experienced were negative for TPOAb and TgAb. With a systematic review of case reports and case series, such cases are not uncommon especially in Japan. The current report might suggest that some proportion of mild to moderate hypothyroidism need to be treated properly, if thyroid autoantibodies were negative in the population at high risk for myxedema coma, such as the elderly and the lean.

## Figures and Tables

**Table 1:** The clinical characteristics and diagnostic score for myxedema coma of the cases.

	TSH (μIU/ml)/fT4 (ng/dl)/fT3 (pg/ml)	Temperature	Neurocognition	GI symptoms	Precipitating event	Cardiovascular dysfunction	Metabolic disturbances	Score
Case 1	34.33/0.6/1.0	<32°C	Somnolent	Absent	Present	Bradycardia (<40 bpm)	Hypercarbia	80
Case 2	40.42/0.1/0.9	32–35°C	Stupor	Absent	Absent	Absent	Hypercarbia	50
Case 3	139.15/0.2/1.4	<32°C	Coma	Absent	Present	Bradycardia (<40 bpm)	Hypercarbia	90

*Note:* A score of 60 or higher is highly suggestive/diagnostic of myxedema coma; a score of 25–59 is suggestive of risk for myxedema coma, and a score below 25 is unlikely to indicate myxedema coma.

Abbreviations: GI, gastrointestinal; TSH, thyroid-stimulating hormone.

**Table 2:** Number of reported cases where thyroid antibodies were available in Japan (including cases in this literature) or other countries.

	Seronegative	Seropositive	Total	*p*
Japan	6 (60%)	4 (40%)	10	0.0192
Other countries	2 (14%)	12 (86%)	14	

*Note*: The *p* value compares the frequency of seronegativity between Japan and other countries using Pearson's chi-square test. For the detail information, see Supporting Information 3: Table S2.

## Data Availability

The data that support the findings of this study are available on request from the corresponding author. The data are not publicly available due to privacy or ethical restrictions.

## Supporting Information

Supporting Information 1 Figure S1: The chest CT of Case 3 on admission.

Supporting Information 2 Table S1: Literatures reporting myxedema coma due to primary hypothyroidism from 1999 to 2022.

Supporting Information 3 Table S2: Number of cases where thyroid antibodies were described from Japan or other countries.
