# Supplementary material for: Frequent Seronegative Primary Hypothyroidism in Myxedema Coma in Japan: Three Case Reports With a Systematic Review
Source: Case Rep Endocrinol. 2024 Oct 14;2024:2524019. doi: 10.1155/2024/2524019 (PMC11493479; doi:10.1155/2024/2524019)
Supplement: Supporting Information 2 — Table S1: Literatures reporting myxedema coma due to primary hypothyroidism from 1999 to 2022. [file 2524019.f2.docx]

Supplementary Table 1. Literature reporting myxedema coma due to primary hypothyroidism from 1999 to 2022. N.D.; not described.

| Year | First author | Country | Number of cases | TPOAb | TgAb | TRAb | Reference |
| --- | --- | --- | --- | --- | --- | --- | --- |
|  | Okuno | **Japan** | 3 | (-) | (-) | (-)(n=2) | this report |
| 2022 | Santos | USA | 1 | (+) | N.D. | N.D. | (1) |
| 2022 | Kaneko | **Japan** | 1 | (-) | (-) | (-) | (8) in the manuscript |
| 2022 | Ito | **Japan** | 1 | (+) | (+) | N.D. | (2) |
| 2022 | Somoza-Cano | USA | 1 | N.D. | N.D. | N.D. | (3) |
| 2022 | Chua | Singapore | 1 | N.D. | N.D. | N.D. | (4) |
| 2021 | Giusti | Italy | 1 | (+) | (+) | (-) | (5) |
| 2021 | Yoshinaka | **Japan** | 1 | (+) | (+) | N.D. | (6) |
| 2021 | Jawed | USA | 1 | (+) | N.D. | N.D. | (7) |
| 2021 | Meling | Norwey | 1 | (+) | (+) | N.D. | (8) |
| 2021 | Gadaen | Netherlands | 2 | N.D. | N.D. | N.D. | (9) |
| 2021 | Elkattawy | USA | 1 | N.D. | N.D. | N.D. | (10) |
| 2021 | Braiteh | USA | 1 | N.D. | N.D. | N.D. | (11) |
| 2021 | Verma | India | 1 | (+) | N.D. | N.D. | (12) |
| 2021 | Izumi | **Japan** | 1 | N.D. | N.D. | N.D. | (13) |
| 2020 | Dixit | USA | 1 | (+) | N.D. | N.D. | (14) |
| 2020 | Toteja | India | 1 | (+) | (+) | N.D. | (15) |
| 2020 | Kousa | USA | 1 | (-) | N.D. | N.D. | (11) in the manuscript |
| 2020 | Roy | India | 10 | N.D. | N.D. | N.D. | (16) |
| 2020 | Acharya | USA | 1 | N.D. | N.D. | N.D. | (17) |
| 2020 | Maldonado | USA | 1 | N.D. | N.D. | N.D. | (18) |
| 2019 | Villalba | Spain | 3 | (+)(n=1) | (-)(n=1) | N.D. | (19) |
| 2019 | Arnautovic | USA | 1 | (+) | N.D. | N.D. | (20) |
| 2019 | Harada | **Japan** | 1 | (-) | (-) | N.D. | (9) in the manuscript |
| 2019 | Mupamombe | USA | 1 | N.D. | N.D. | N.D. | (21) |
| 2019 | Yafit | Israel | 3 | N.D. | N.D. | N.D. | (22) |
| 2018 | Gocho | **Japan** | 1 | (+) | (+) | (-) | (23) |
| 2018 | Sethi | India | 1 | N.D. | N.D. | N.D. | (24) |
| 2017 | Kirsch | Switzerland | 1 | (+) | N.D. | N.D. | (25) |
| 2017 | Salhan | USA | 1 | N.D. | N.D. | N.D. | (26) |
| 2017 | Gunatilake | Sri Lanka | 1 | N.D. | N.D. | N.D. | (27) |
| 2017 | Batista | USA | 1 | N.D. | N.D. | N.D. | (28) |
| 2016 | Patel | USA | 1 | N.D. | N.D. | N.D. | (29) |
| 2015 | Dhakal | USA | 1 | (-) | N.D. | N.D. | (12) in the manuscript |
| 2015 | Majid-Moosa | USA | 1 | N.D. | N.D. | N.D. | (30) |
| 2015 | Dixit | India | 1 | N.D. | N.D. | N.D. | (31) |
| 2014 | Popoveniuc | USA | 12 | N.D. | N.D. | N.D. | (4) in the mauscript |
| 2012 | Yanamandra | India | 1 | (+) | N.D. | N.D. | (32) |
| 2012 | Baduni | India | 1 | N.D. | N.D. | N.D. | (33) |
| 2012 | Komiya | **Japan** | 1 | N.D. | N.D. | N.D. | (34) |
| 2012 | Kitamura | **Japan** | 1 | (-) | (-) | (-) | (10) in the manuscript |
| 2011 | Kogan | Israel | 1 | N.D. | N.D. | N.D. | (35) |
| 2011 | Mallipedhi | UK | 1 | (+) | N.D. | N.D. | (36) |
| 2010 | Ahn | Korea | 1 | N.D. | N.D. | N.D. | (37) |
| 2010 | Kargili | Turkey | 1 | N.D. | N.D. | N.D. | (38) |
| 2009 | Lee | USA | 1 | N.D. | N.D. | N.D. | (39) |
| 2008 | Dutta | India | 18 | N.D. | N.D. | N.D. | (40) |
| 2007 | Taguchi | **Japan** | 1 | (+) | (+) | (+) | (41) |
| 2007 | Cappelli | Italy | 1 | N.D. | N.D. | N.D. | (42) |
| 2007 | Sheu | Taiwan | 1 | N.D. | N.D. | N.D. | (43) |
| 2004 | Rodriguez | Spain | 8 | N.D. | N.D. | N.D. | (3) in the mauscript |
| 2001 | Doherty | USA | 1 | N.D. | N.D. | N.D. | (44) |
| 1999 | Yamamoto | **Japan** | 8 | N.D. | N.D. | N.D. | (45) |
|  |  |  |  |  |  |  |  |
|  | Total |  | 111 |  |  |  |  |

Reference.

**1.** Santos Argueta A, Doukas SG, Roy R. New-Onset Hypothyroidism Manifesting As Myxedema Coma: Fighting an Old Enemy. Cureus 2022; 14:e23881

**2.** Ito H, Fukuda K, Ashida K, Nagayama A, Sako T, Mizuochi K, Kabashima M, Yoshinobu S, Iwata S, Hasuzawa N, Hayashi S, Akashi T, Nomura M. Case Report: Myxedema Coma Caused by Immunoglobulin A Vasculitis in a Patient With Severe Hypothyroidism. Front Immunol 2022; 13:838739

**3.** Somoza-Cano FJ, Al Armashi AR, Patell K, Hammad F, Ravakhah K. Status Epilepticus as a Life-Threatening Manifestation of Myxedema Crisis. Cureus 2022; 14:e21155

**4.** Chua MWJ. A Patient with Recurrent Myxedema Coma: What Was the Missing Link? Am J Med 2022; 135:393-396

**5.** Giusti M, Maio A. Acute thyroid swelling with severe hypothyroid myxoedema after COVID-19 vaccination. Clinical case reports 2021; 9:e05217

**6.** Yoshinaka A, Akatsuka M, Yamamoto S, Yamakage M. Sudden cardiac arrest associated with myxedema coma due to undiagnosed hypothyroidism: a case report. BMC Endocr Disord 2021; 21:229

**7.** Jawed M, Osella J, Bani Hani D. A Case of Myxedema Coma Crisis Induced by Inhalation Injury. Cureus 2021; 13:e17049

**8.** Meling Stokland AE, Dahle AL, Kloster VL, Nedrebø T, Nedrebø BG. Myxedema coma complicated by bilateral hygromas. Endocrinology, diabetes & metabolism case reports 2021; 2021

**9.** Gadaen RJ, Tummers-de Lind van Wijngaarden RF. Hypothyroid Crisis: Oral or Intravenous Treatment? A Report of Two Cases. European journal of case reports in internal medicine 2021; 8:002752

**10.** Elkattawy S, Dhanoa P, Kotys J, Fichadiya H, Eckman A. Myxedema Coma: Case Report and Literature Review. Cureus 2021; 13:e15277

**11.** Braiteh N, Senyondo GD, Rahman MF, Chaudhry R, Kashou H. An Unusual Presentation of ST Elevation Myocardial Infarction Complicated with Cardiogenic Shock Due to Myxedema Coma: A Case Report. The American journal of case reports 2021; 22:e929573

**12.** Verma V, Menon AS, Bahadur A, Sikarwar A. Clinical manifestation and images of a patient having cardiovascular involvement in myxoedema coma. Medical journal, Armed Forces India 2021; 77:92-95

**13.** Izumi K, Ono Y. [A case of elderly myxedema coma patient with end-stage renal failure]. Nihon Ronen Igakkai Zasshi 2021; 58:152-157

**14.** Dixit NM, Truong KP, Rabadia SV, Li D, Srivastava PK, Mosaferi T, Calfon Press MA, Donangelo I, Kelesidis T. Sudden Cardiac Arrest in a Patient With Myxedema Coma and COVID-19. Journal of the Endocrine Society 2020; 4:bvaa130

**15.** Toteja N, Khera D, Sasidharan R, Choudhary B, Singh K. Enteric Fever Precipitating Myxedema Crisis. Indian J Pediatr 2020; 87:873-874

**16.** Roy N, Majumder A, Sanyal D, Chaudhuri SR, Sarkar S, Pathak A. Legions of Presentations of Myxedema Coma: A Case Series from a Tertiary Hospital in India. Journal of the ASEAN Federation of Endocrine Societies 2020; 35:233-237

**17.** Acharya R, Cheng C, Bourgeois M, Masoud J, McCray E. Myxedema Coma: A Forgotten Medical Emergency With a Precipitous Onset. Cureus 2020; 12:e10478

**18.** Maldonado D, Patel U, Tarlin N. A Case of Refractory Myxedema Coma. Cureus 2020; 12:e9737

**19.** Villalba NL, Zulfiqar AA, Saint-Mezard V, Ortiz MBA, Kechida M, Zamorano NF, Ortega SS. Myxedema coma: four patients diagnosed at the Internal Medicine Department of the Dr. Negrin University Hospital in Spain. Pan Afr Med J 2019; 34:7

**20.** Arnautovic JZ, Connor-Schuler R, Ip R. Mechanical Circulatory Support in Management of Cardiogenic Shock and Myxedema Coma. Case reports in cardiology 2019; 2019:2595736

**21.** Mupamombe CT, Reyes FM, Laskar DB, Gorga J. Myxedema Coma Complicated by Pancytopenia. Case Rep Med 2019; 2019:2320751

**22.** Yafit D, Carmel-Neiderman NN, Levy N, Abergel A, Niv A, Yanko-Arzi R, Zaretski A, Wengier A, Fliss DM, Horowitz G. Postoperative myxedema coma in patients undergoing major surgery: Case series. Auris Nasus Larynx 2019; 46:605-608

**23.** Gocho N, Aoki E, Okada C, Hirashima T. Myxedema Coma Following the Administration of Gonadotropin-releasing Hormone Agonist Complicated by Acute Pancreatitis. Intern Med 2018; 57:3117-3122

**24.** Sethi P, Kamal M, Verma S, Bhatia P. Undiagnosed myxedema coma: Rare but life-threatening. Saudi J Anaesth 2018; 12:173-174

**25.** Kirsch M, Rimpau C, Nickel CH, Baier P. Intracerebral Bleeding and Massive Pericardial Effusion as Presenting Symptoms of Myxedema Crisis. Case reports in emergency medicine 2017; 2017:8512147

**26.** Salhan D, Sapkota D, Verma P, Kandel S, Abdulfattah O, Lixon A, Zwenge D, Schmidt F. Sudden cardiac arrest as a rare presentation of myxedema coma: case report. Journal of community hospital internal medicine perspectives 2017; 7:318-320

**27.** Gunatilake SSC, Bulugahapitiya U. Myxedema Crisis Presenting with Seizures: A Rare Life-Threatening Presentation-A Case Report and Review of the Literature. Case reports in endocrinology 2017; 2017:4285457

**28.** Batista AS, Zane LL, Smith LM. Burn-induced Myxedema Crisis. Clinical practice and cases in emergency medicine 2017; 1:98-100

**29.** Patel P, Bekkerman M, Varallo-Rodriguez C, Rampersaud R. An Atypical Case of Myxedema Coma with Concomitant Nonconvulsive Seizure. Case reports in critical care 2016; 2016:3438080

**30.** Majid-Moosa A, Schussler JM, Mora A. Myxedema coma with cardiac tamponade and severe cardiomyopathy. Proc (Bayl Univ Med Cent) 2015; 28:509-511

**31.** Dixit S, Dutta MK, Namdeo M. A Rare Case of Myxedema Coma with Neuroleptic Malignant Syndrome (NMS). Journal of clinical and diagnostic research : JCDR 2015; 9:Vd01-vd03

**32.** Yanamandra U, Kotwal N, Menon A, Nair V. Ogilvie's syndrome in a case of myxedema coma. Indian J Endocrinol Metab 2012; 16:447-449

**33.** Baduni N, Sinha SK, Sanwal MK. Perioperative management of a patient with myxedema coma and septicemic shock. Indian J Crit Care Med 2012; 16:228-230

**34.** Komiya A, Watanabe A, Kawauchi Y, Takano A, Fuse H. Severe oligozoospermia in a patient with myxedema coma. Reprod Med Biol 2012; 11:207-211

**35.** Kogan A, Kassif Y, Shadel M, Shwarz Y, Lavee J, Or J, Raanani E. Severe hypothermia in myxoedema coma: a rewarming by extracorporeal circulation. Emerg Med Australas 2011; 23:773-775

**36.** Mallipedhi A, Vali H, Okosieme O. Myxedema coma in a patient with subclinical hypothyroidism. Thyroid 2011; 21:87-89

**37.** Ahn JY, Kwon HS, Ahn HC, Sohn YD. A case of myxedema coma presenting as a brain stem infarct in a 74-year-old Korean woman. J Korean Med Sci 2010; 25:1394-1397

**38.** Kargili A, Turgut FH, Karakurt F, Kasapoglu B, Kanbay M, Akcay A. A forgotten but important risk factor for severe hyponatremia: myxedema coma. Clinics (Sao Paulo, Brazil) 2010; 65:447-448

**39.** Lee CH, Wira CR. Severe angioedema in myxedema coma: a difficult airway in a rare endocrine emergency. Am J Emerg Med 2009; 27:1021.e1021-1022

**40.** Dutta P, Bhansali A, Masoodi SR, Bhadada S, Sharma N, Rajput R. Predictors of outcome in myxoedema coma: a study from a tertiary care centre. Crit Care 2008; 12:R1

**41.** Taguchi T, Iwasaki Y, Asaba K, Takao T, Hashimoto K. Myxedema coma and cardiac ischemia in relation to thyroid hormone replacement therapy in a 38-year-old Japanese woman. Clin Ther 2007; 29:2710-2714

**42.** Cappelli C, Stanga B, Paini A, Gandossi E, Cumetti D, Castellano M, Muiesan ML, Agabiti Rosei E. Myxoedema coma precipitated by diabetic ketoacidosis and neuroleptic drugs: case report in an intensive care unit. Intern Emerg Med 2007; 2:147-149

**43.** Sheu CC, Cheng MH, Tsai JR, Hwang JJ. Myxedema coma: a well-known but unfamiliar medical emergency. Thyroid 2007; 17:371-372

**44.** Doherty MJ, Baxter AB, Longstreth WT, Jr. Herpes simplex virus encephalitis complicating myxedema coma treated with corticosteroids. Neurology 2001; 56:1114-1115

**45.** Yamamoto T, Fukuyama J, Fujiyoshi A. Factors associated with mortality of myxedema coma: report of eight cases and literature survey. Thyroid 1999; 9:1167-1174
