# Supplementary material for: Frequent Seronegative Primary Hypothyroidism in Myxedema Coma in Japan: Three Case Reports With a Systematic Review
Source: Case Rep Endocrinol. 2024 Oct 14;2024:2524019. doi: 10.1155/2024/2524019 (PMC11493479; doi:10.1155/2024/2524019)
Supplement: Supporting Information 3 — Table S2: Number of cases where thyroid antibodies were described from Japan or other countries. [file 2524019.f3.docx]

|  | Described | Not described | Total | p |
| --- | --- | --- | --- | --- |
| Japan | 10 (50%) | 10 (50%) | 20 | 0.0006 |
| Other countries | 14 (15%) | 77 (85%) | 91 |  |

Supplementary Table 2. Number of cases where thyroid antibodies were described from Japan (including cases in this literature) or other countries. The p-value compares the frequency of described cases between Japan and other countries using Pearson’s chi-square test. For the detail information, see Supplementary Table 1.
